# Out-coupling of Longitudinal Photoacoustic Pulses by Mitigating the Phase Cancellation

**DOI:** 10.1038/srep21511

**Published:** 2016-02-12

**Authors:** Taehwa Lee, Qiaochu Li, L. Jay Guo

**Affiliations:** 1 Department of Mechanical Engineering, University of Michigan, Ann Arbor, MI48109, USA; 2 Department of Electrical Engineering and Computer Science, University of Michigan, Ann Arbor, MI48109, USA

## Abstract

Waves of any kinds, including sound waves and light waves, can interfere constructively or destructively when they are overlapped, allowing for myriad applications. However, unlike continuous waves of a single frequency, interference of photoacoustic pulses is often overlooked because of their broadband characteristics and short pulse durations. Here, we study cancellation of two symmetric photoacoustic pulses radiated in the opposite direction from the same photoacoustic sources near a free surface. The cancellation occurs when one of the two pulses is reflected with polarity reversal from the free surface and catches up with the other. The cancellation effect, responsible for reduced signal amplitudes, is systematically examined by implementing a thin transparent matching medium of the same acoustic impedance. By changing the thickness of the transparent layer, the overlap of the two symmetric pulses is controlled. For optimized matching layers, the cancellation effect can be significantly reduced, while the resulting output waveform remains unchanged. Similar to the planar absorber, different dimensional absorbers including cylinders and spheres also exhibit the cancellation between the outward and inward waves. This work could provide further understanding of photoacoustic generation and a simple strategy for increasing photoacoustic signal amplitudes.

Research on the photoacoustic (PA) effect, resulting from the absorption of electromagnetic radiation^1^,^2^, has been motivated by various applications ranging from biomedical imaging^3^,^4^, non-destructive test and evaluation (NDT and NDE)^5^, to more recently, THz optoacoustic detection^6^, and the study of the heat transport^7^. For those applications, knowing the amplitude dependence of the PA effect is essential for the interpretation of experimental data. Also, this knowledge could be used to increase photoacoustic amplitudes for applications, e.g., photoacoustic imaging and photoacoustic cavitation generation^8^,^9^. To obtain high-amplitude photoacoustic signals, focus has been typically placed on choosing materials with good light absorption and high thermal expansion coefficient, or simply increasing incident light intensity. However, for given materials, their light-induced damages determine the upper-limit of the achievable photoacoustic signals.

Resonance phenomena due to the constructive interference of waves are often exploited to produce high-amplitude waves. However, the resonances are frequency dependent such that it is difficult to implement for broadband photoacoustic pulses. Also, the short photoacoustic pulses make it even harder to meet the resonance requirement for the spatial and temporal overlap of the waves. Despite such difficulties, the interference of photoacoustic pulses cannot be ignored. In fact, the interference effect could be important for the actual photoacoustic generation, which was supported by work done by Diebold *et al.*^10^,^11^,^12^. To study photoacoustic monopole radiation in two and three dimensions, the authors considered two symmetric photoacoustic waves oppositely radiated from the same photoacoustic sources. In the study, one of the two is reflected with a phase shift and overlaps with the other. However, little attention has been paid to a significant change in photoacoustic amplitudes by the temporal overlap of the two pulses. Furthermore, the individual contribution of the two propagating waves is rarely identified in the measured waveform. This is because the two waves are very close to each other such that the resulting photoacoustic wave is represented by the time-derivative of each wave. Besides two and three dimensions, photoacoustic waves in one dimensional absorbers for an air-backed boundary (or pressure-release boundary) is also expressed by the time-derivative form^13^,^14^, suggesting that the interaction of two photoacoustic pulses could play an important role.

The reduced longitudinal pressure amplitude in metal absorbers subject to an air-backed boundary was reported elsewhere^15^,^16^,^17^, where the significant reduction was explained by absence of normal stress components at the free surface. Although some studies suggested that the reduced longitudinal pressure amplitude may result from acoustic interference^18^, approaches to increasing longitudinal pressure amplitudes mostly relied on burying photoacoustic sources under thick transparent materials for strong normal loading, rather than controlling the interference effect investigated in this work. Since the mechanical properties of the thick transparent materials are much different from those of metals, the destructive interference of the two symmetric pulses cannot be effectively prevented. Instead, the significant enhancements reported were likely achieved due to other effects including thermoelastic generation and explosive phase change of the liquid cover layer heated by the adjacent light-absorbing metals. Also, the transparent coverings had thickness much larger than the spatial extent of acoustic pulses (or acoustic wavelength) such that photoacoustic pulses reflected from the air-backed boundary are far separated from the other outgoing waves. Although a detailed study on the interference effect of photoacoustic pulses could be valuable, it would be difficult to find transparent materials of the same mechanical properties as metals.

In this work, we study the temporal overlap of the two symmetric photoacoustic pulses produced by the same photoacoustic sources in the vicinity of sound reflecting boundaries. Significant amplitude cancellation is observed when one of the two pulses is reflected with polarity reversal from a pressure-release boundary and overlaps with the other pulse in space and time. The cancellation effect is demonstrated by using a polymer light absorber buried by a thin water layer, whose thickness is comparable to the acoustic wavelengths. Owing to negligible acoustic reflection between the water layer and the polymer absorber, the photoacoustic pulse propagating across the water layer has a sufficient time delay, leading to reduced overlap between the two pulses with opposite polarity and thereby increased amplitude. Not only can the cancellation be effectively mitigated, but also the output waveform remains the same, which is in a sharp contrast to a significant change in waveforms for approaches using thick impedance-mismatched coverings. Furthermore, the cancellation effect for the planar absorber is extended to different dimensional absorbers, such as cylinders and spheres, providing further understanding of photoacoustic generation.

## Results and Discussion

### Phase cancellation in one-dimensional (1D) absorber

**Figure 1:**
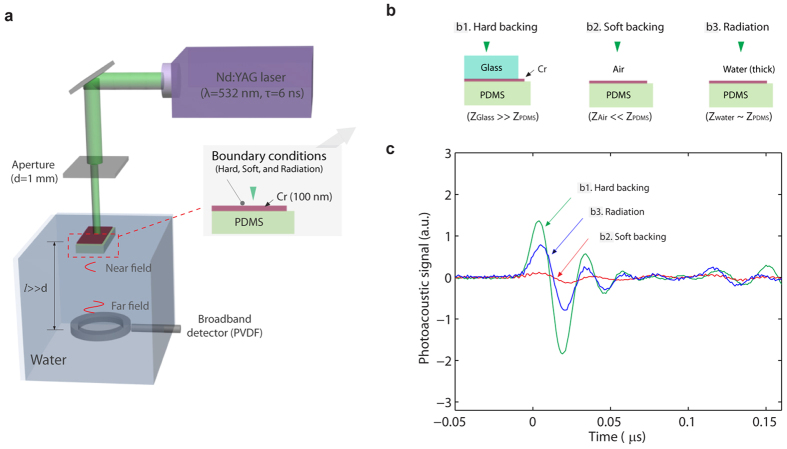
(**a**) Experimental setup for photoacoustic generation and measurement. A 6-ns pulsed laser beam is illuminated through an aperture (1 mm in diameter) to samples consisting of a metal layer (Cr 100 nm) and an adjacent PDMS layer. Photoacoustic signals are measured by a broadband PVDF detector in the far field (the distance between the samples and the detector is 15 mm; the laser spot size is 1 mm). (**b**) Samples for the different boundary conditions using different backing materials: Glass for hard boundary, air for soft boundary, water for radiation boundary. The boundary conditions differ based on the acoustic impedance difference between backing materials and the PDMS layers because the thin metal layer is acoustically transparent (its thickness is much smaller than the acoustic wavelength); glass for hard boundary (
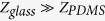
), air for soft boundary (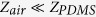), and water for radiation boundary (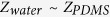). For radiation boundary, no significant reflection at the water/PDMS interface occurs. (**c**) The measured photoacoustic signals for the different boundary conditions.

Photoacoustic (PA) generation is studied for different acoustic boundary conditions. As the experimental setup is illustrated in Fig. 1(a), a pulsed laser beam is illuminated on samples consisting of a thin metal layer and an adjacent polydimethylsiloxane (PDMS) elastomer layer. Here, the metal layer functions as a light absorbing medium that converts absorbed optical energy into thermal energy, while the PDMS layer serves as a substrate for metal deposition. As shown in Fig. 1(b), we apply three acoustic boundary conditions by covering the metal layer with different backing mediums: Glass for hard boundary, air for soft boundary, and water for radiation boundary (i.e., no acoustic reflection occurs). Note that the thickness of the metal absorbers (100 nm) are much smaller than the acoustic pulse wavelength (>10 μm) so that the boundary conditions differ based on the acoustic impedance difference between the backing materials and the PDMS layers, not between the baking materials and the thin metal absorber. PA signals are measured for the different boundary conditions, as shown in Fig. 1(c). Despite the same light absorbing layer, the generated PA signals vary a lot with the boundary conditions. The hard boundary shows the highest signal amplitude, while the amplitude for the radiation boundary is only half the amplitude. Most notably, the soft boundary case produces significantly low amplitude, which is an order of magnitude lower than the others. Also, the waveform for the soft boundary is different from the others.

The difference in the waveforms has been observed and discussed elsewhere^13^,^19^. Also, the significant reduction in longitudinal pressure amplitudes is understood as the absence of normal stress components, which is addressed by burying photoacoustic sources under thick transparent coverings^15^,^16^,^17^. Although some studies suggested that destructive interference is the reason for the reduced amplitude^18^, methods for enhancing pressure amplitudes are not based on elimination of the interference. Instead, besides strong normal loading, explosive evaporation and thermoelastic generation in liquid cover layers are considered to be a main factor for the enhancement. In the studies, the transparent coverings have mechanical properties much different from those of the light absorbers (e.g., metals), and thus the covering approaches are ineffective in removing the destructive interference because of the significant acoustic reflection between the transparent coverings and metals (see Supplementary Note 1 online).

**Figure 2:**
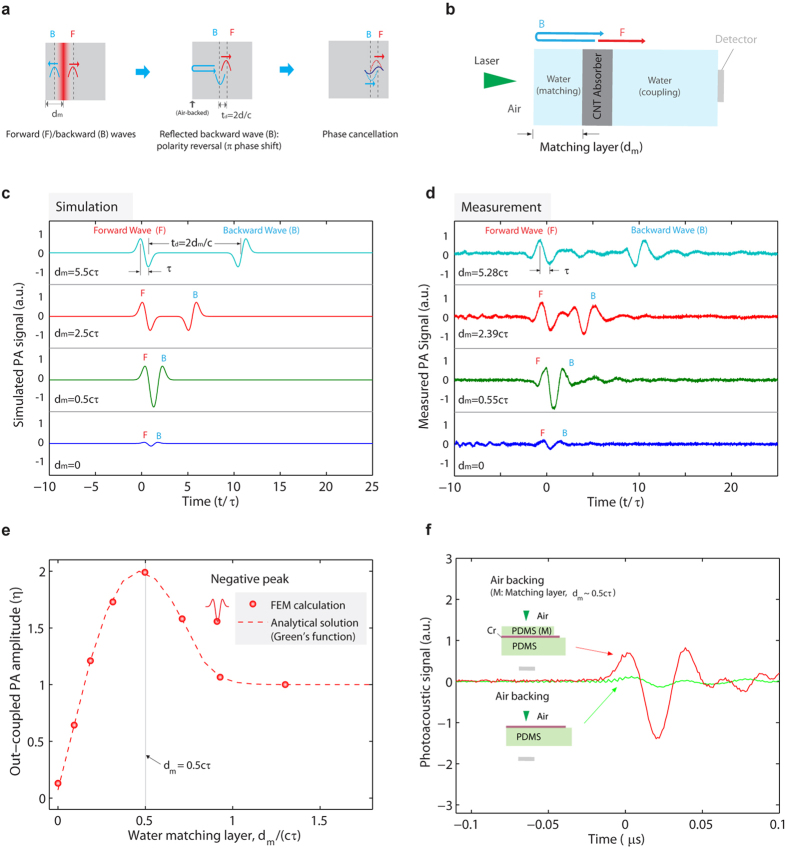
(**a**) The schematics of the photoacoustic (PA) cancellation. Pulsed optical heating simultaneously produces two oppositely propagating waves (F, forward wave; B, backward wave). The backward wave is reflected with a π phase shift (or polarity reversal) from the air-backed interface (B), immediately following the original forward-going wave and thus cancelling each other. (**b**) The cancellation effect is experimentally demonstrated by using a polymer light absorber composed of PDMS and carbon nanotube fillers. PA signals are recorded with varying the thickness of the water layer on the air (i.e., water matching layer). Since the acoustic impedance of PDMS is similar to that of water, no significant reflection between the PDMS-based absorber and the water layer occurs. (**c**) Calculated PA signals for different water matching layer thicknesses (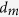). (**d**) Measured PA signals. (**e**) The out-coupled photoacoustic amplitude (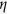) for different water matching layer thickness, defined by a ratio of the peak negative amplitude (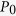) to the peak negative amplitude for radiation boundary (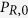), i.e., 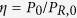. The maximum out-coupled photoacoustic amplitude arises at 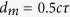. (**f**) Measured PA signals for the soft boundary with and without a PDMS matching layer. The PA amplitude for the sample with the matching layer is an order of magnitude larger than that for without the matching layer.

The interference effect of the two symmetric PA pulses is theoretically investigated by controlling the temporal overlap of the two, with an aim of explaining the reason for the reduced pressure amplitude and thus increasing PA pressure amplitudes. As illustrated in Fig. 2(a), upon optical excitation, the thin absorber layer launches two oppositely propagating PA pulses. The backward wave experiences a π phase shift when reflecting from the air-backed boundary. As the π phase shift occurs for waves of all frequencies, the reflected broadband pulse reverts its waveform in time, i.e., the amplitude flips its sign. Therefore the reflected pulse acquires the opposite polarity and can partially cancel the original forward wave, depending on the overlap between the two pulses. Ideally, control of the overlap between the two pulses is enabled by using a transparent covering medium that has the same mechanical properties as photoacoustic sources. By changing the thickness of the transparent medium, the interaction of the two pulses is simulated, as the simulation result is shown in Fig. 2(c) (see Supplementary Note 2 online for detailed calculation). With increasing the thickness of the transparent covering, the temporal separation of the reflected pulse and the original forward pulse is enlarged. For large temporal separation, the two with the opposite polarity propagate independently (the top two curves). However, if the temporal separation is sufficiently small, the reflected pulse overlaps with the forward pulse, leading to significant amplitude cancellation. An extreme case is shown as the blue curve in Fig. 2(c), where the two pulses almost completely cancel each other, resulting in a drastically reduced wave amplitude.

The ideal situation proposed in the simulation is experimentally demonstrated by using a water covering and a polymer light absorber consisting of carbon nanotube (CNT) fillers embedded in an elastomeric material PDMS, as shown in Fig. 2(b). Because of its acoustic impedance matched to the polymer absorber, the water layer can minimize unwanted acoustic reflection between the water layer and the polymer, while providing an effective means to control the time delay of the backward pulse reflected from the air boundary. Here, we will refer the water layer as matching layer. By varying the water matching layer thickness (*d*_m_), the PA signals are measured, as shown in Fig. 2(d). The experimental result shows an excellent agreement with the simulation result. Specifically, it is observed that the reflected pulse has the opposite polarity and a delay time corresponding to the acoustic transit time across the water matching layer (i.e., 2*d*_m_/*c*). Based on the interaction of the two PA pulses, it is easily understood that PA amplitude for the radiation boundary is only half that for the hard boundary; the backward pulse for the radiation boundary does not interact with the forward pulse, whereas for the hard boundary the backward pulse with the same polarity constructively interferes with the forward pulse. Also, it is interesting to note that by properly choosing the matching layer, the two pulses merge into a single pulse with large negative amplitude (green curve).

### Out-coupling of photoacoustic pulses

The matching layer on the air side allows for “out-coupling” of the two symmetric PA pulses, which otherwise cancel out in light absorbers. The thin matching layer is used to effectively provide a time delay to the pulse propagating through the matching layer. However, the boundary on the air side of the matching layer works as a total reflection interface, there is no PA pulse propagating out to the air. Thus, the matching layer used here is a lot different from the impedance matching layers used for piezoelectric transducers, which are inserted between the piezoelectric elements and coupling mediums so as to maximize the output of the acoustic energy transfer through the coupling mediums. Although the optimization of the matching layers for piezoelectric transducers is based on acoustic interference, the interference of a single pulse that occurs between two reflecting boundaries is totally different from the interference of the two symmetric PA pulses radiated from the same PA sources.

The out-coupling of PA pulses suggests that we can define out-coupled pressure amplitudes, normalized to the peak amplitude for the radiation boundary (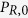), i.e., 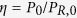, where 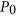 is the peak negative amplitude. The out-coupled pressure amplitude (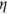) is plotted as a function of matching layer thickness, as shown in Fig. 2(e). The symbols indicate the values obtained by the FEM calculation dealing with the thermoelastic generation, while the dashed line represents those calculated by the one-dimensional (1D) analytical solution using the Green’s function approach (see Supplementary Note 3 online for details). As can be seen in the figure, the two different calculations show an excellent agreement. Since the Green’s function approach implements two symmetric pulses to calculate the resulting PA amplitudes, the excellent agreement indicates that the interference of the two PA pulses indeed determines the output PA amplitudes. Also, in the curve, peak out-coupling is observed at a certain matching layer thickness due to constructive interference, which cannot be explained by static normal loading. The maximum out-coupling occurs at a matching layer thickness of *d*_m_ = 0.5*cτ* (*τ* is the PA pulse duration for the radiation boundary, defined as the peak-to-peak time). For the optimum thickness, the reflected pulse has the right time delay such that the negative peaks of the two pulses are exactly overlapped, as also can be seen in Fig. 2(c,d). It is worthy to note that the resulting negative amplitude becomes comparable to that for the hard boundary. We should also mention that as the optimum out-coupling depends on the PA pulse duration, both the bandwidths of detectors and acoustic attenuation that can change the measured pulse duration have a considerable effect on the optimum out-coupling.

Instead of evaporative water, by using a permanent coating, e.g., PDMS layer, the optimum out-coupling is demonstrated for the metal-PDMS structures in Fig. 1. By adding a PDMS matching layer of approximately 10 μm, the signal amplitude for the soft boundary is significantly enhanced. As shown in Fig. 2(f), the quasi-monopolar waveform with increased negative amplitude is measured. It is interesting to note that despite the addition of the thin PDMS matching layer, the waveform remains the same, which is very different from substantial changes in waveforms for thick transparent coverings^17^. Originally, PA waveforms for the soft boundary are quasi-monopolar because of the immediate overlap between the two PA pulses with the opposite polarity. Unlike the thick coverings, the matching layer is still thin enough to allow for the overlap of the two PA pulses. This result suggests that the matching layer approach could provide with tunability of PA waveforms, which could be useful for waveform-sensitive applications, e.g., PA cavitation and imaging^9^,^20^,^21^.

The matching layer approach can be widely applicable to cases where air-backed interfaces arise. Specifically, polymer-based light absorbers consisting of carbon fillers have been recently reported for efficient PA generation by taking advantage of their high thermal expansion^22^,^23^,^24^,^25^. For the polymer absorbers, Several transparent matching mediums could be available, thus allowing to implement the matching approach. For example, in a recent work in our lab, a polymer light absorber for terahertz detection is required to be in form of a free-standing film, because adjacent hard materials can either block sound waves or terahertz waves^6^. By using thin matching layer approach for enhanced PA generation, we could anticipate a considerable increase in detection sensitivity. On the other hand, in laser ultrasonics, the matching layer approach might not be practical, because choosing the right matching medium would be challenging, especially for metals. However, our study could be beneficial in assessing a possible reduction in the interference effects by transparent coverings. Also, as thick transparent coverings on metals are not effective in eliminating the cancellation effect, we can suggest that to mitigate the cancellation, the acoustic properties of transparent coverings should be similar to those of metals, especially when thermoelastic generation and explosive phase change in the coverings are negligible for low optical energies.

### Effect of optical penetration depth

**Figure 3:**
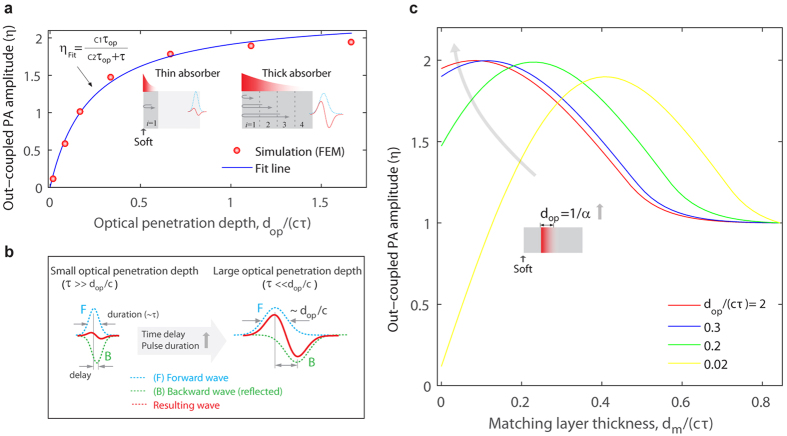
(**a**) The out-coupled photoacoustic amplitude (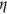) versus optical penetration depth (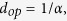) for no matching layer. Also plotted is a fit line obtained by a ratio of the time delay (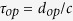) to the acoustic pulse duration 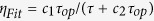 where the fitting parameters 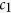 and 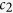 are 10.5 and 4.5, respectively. The inset shows the schematics of the PA out-coupling for different optical penetration depths. For thick absorbers, large PA out-coupling results from the combined contribution of many layers virtually divided. (**b**) Illustration of the effect of the optical penetration depth on the PA out-coupling. Both the time delay and acoustic pulse duration increase with the optical penetration depth. For large optical penetration depths (or the short pulse regime; 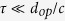), the time delay is comparable to the acoustic pulse duration, thus minimizing the cancellation effect. The dashed lines indicates the forward and backward waves that are overlapped, while the solid line represents the resulting wave. (**c**) The out-coupled photoacoustic amplitude versus matching layer thickness for different optical penetration depths. The matching layer approach is more effective in smaller optical penetration depths.

According to the discussion above, if there is no time delay between the two photoacoustic (PA) pulses simultaneously produced, complete cancellation should occur, leading to zero amplitude. However, this complete cancellation was not observed in the experiment or in simulation. This means that without matching layer, a non-negligible time delay should exist between the two pulses that hinders the total cancellation. Indeed, there is an intrinsic time delay, which is related to the optical penetration depth (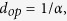
where 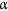 is the light absorption coefficient). The intrinsic time delay 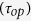 is found to correspond to the acoustic transit time across the optical penetration depth, i.e., 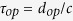 (see Supplementary Note 4 online for more discussion). As matching-layer-induced time delay allows enhanced out-coupling of PA pulses, the intrinsic time delay due to the optical penetration depth can show similar effects on the PA out-coupling. As shown in Fig. 3(a), the out-coupling amplitude increases with the optical penetration depth. In other word, mediums with strong light absorption (or smaller optical penetration depths 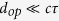) have low out-coupling amplitudes and significant cancellation effect.

Besides the time delay, the acoustic pulse duration can influence the interaction of the PA pulses. Unlike the matching layer that can only change the time delay, the optical penetration depth can broaden the acoustic pulse duration. The effect of the broadened acoustic pulse duration can be seen in Fig. 3(a), where the out-coupling amplitude is saturated at large optical penetration depths 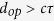. The saturation is because the broadened acoustic pulse duration can prevent from complete separation of the two PA pulses, as illustrated in Fig. 3(b). For large optical penetration depths or the short pulse regime (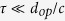), the acoustic pulse duration is comparable to the time delay between the two pulses, thus maximizing the out-coupling efficiency. In contrast, for small optical penetration depths or the long pulse regime (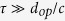), the time delay is much shorter than the acoustic pulse duration. Figure 3(a) also shows a fit line calculated as 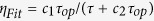. The fit line is calculated as a ratio of the intrinsic time delay 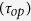 to the acoustic pulse duration, which is suggested by the derived out-coupling efficiency for the long pulse regime 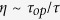, where 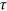 is the acoustic pulse duration for the long pulse regime (see Supplementary Note 5 online for the detailed derivation). The denominator is modified to take into account the broadened acoustic pulse duration due to the optical penetration depth. The fit line shows a good agreement with the simulation result, again confirming that the PA out-coupling is influenced by both the time delay and the acoustic pulse duration.

The matching layer approach is more effective in smaller optical penetration depths, as shown in Fig. 3(c). The cancellation effect will be minimized for weakly absorbing mediums, such as liquids and polymers. Thus, the matching layer approach does not show significant improvement. However, the cancellation is detrimental to the output acoustic amplitude when light absorption is highly confined near the interface, e.g., metal absorbers irradiated by a nanosecond pulsed laser. In these cases, adding the matching layer will effectively mitigate the cancellation and thus increase signal amplitude.

### Phase cancellation in different dimensions: spherical and cylindrical objects

**Figure 4:**
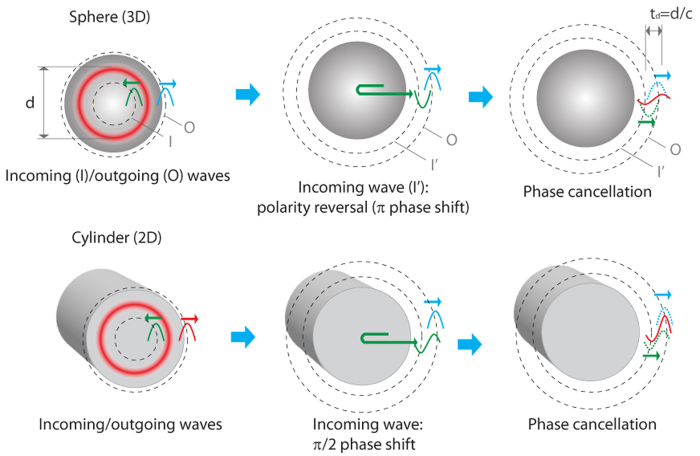
Illustration of the phase cancellation in cylindrical and spherical objects. Two oppositely propagating waves (I: inward and O: outward) are produced by pulsed symmetric heating. As going through the center, the inward wave experiences a phase shift, then immediately following the original outward wave. For the spherical wave, a π phase shift (or polarity reversal) occurs, while for the cylindrical wave, a π/2 phase shift (bipolar waveform) occurs. The waves with the phase shifts can interfere with the other waves, which depends on the delay time between the two waves.

**Figure 5:**
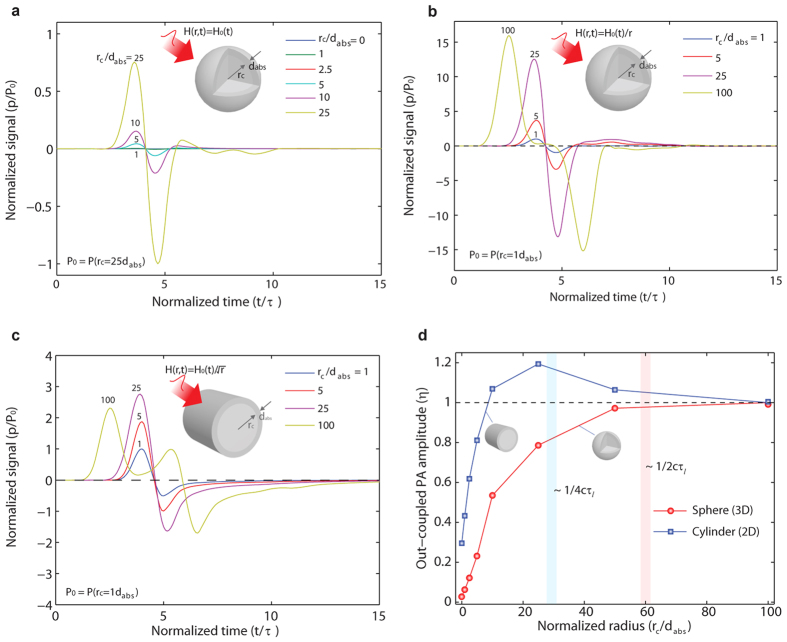
(**a**) Simulated photoacoustic (PA) signals of a sphere with a transparent core (*r*_*c*_) and an absorbing shell (*d*_*abs*_). The transparent core serves as an impedance matching medium analogous to the matching layer for the planar absorber. PA signals are produced by the spatially uniform heating *H*_0_(*t*), which is the Gaussian temporal pulse with pulse duration *τ*. The PA signals for different matching core radii are obtained at a distance of *r*_obs_ from the center. And the signals are normalized to the pressure amplitude for a sphere with *r*_c_ = 1 *d*_abs_, i.e., P_0_ = P(*r*_c_ = 1 *d*_abs_). The resulting signals are due to the combined effect of the PA cancellation and 1/*r*-decay of spherical waves. (**b**) PA signals for the scaled heating function *H*_0_(*t*)/*r* to only evaluate the cancellation effect. The two PA pulses are completely separated at *r*_c_ = 100 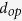. (**c**) Simulated PA signals for a cylinder subject to the scaled heating function *H*_0_(*t*)/*r*^0.5^ (1/ *r*^0.5^-decay of cylindrical waves). (**d**) The out-coupled photoacoustic amplitude (*η*) versus the core radius (*r*_c_) for spheres and cylinders with the constant shell thickness (*d*_abs_).

The cancellation effect observed in the 1D absorbers with the air-backed interface can be similarly observed in spherical and cylindrical objects, where radially symmetric optical heating yields two oppositely propagating waves (inwardly and outwardly)^12^. As illustrated in Fig. 4, the inwardly propagating wave can experience phase shifts depending on dimension, known as Gouy phase shift (π phase shift for a spherical wave, π/2 phase shift for a cylindrical wave)^26^. The waves with the phase shifts can interfere with the original outgoing waves. If dispersion can be neglected, a pulse comprising of waves of a range of frequencies will follow similar phase shift, and interact with the original outgoing pulse when they are temporally overlapped. To control the interaction of the two waves, transparent matching cores (analogous to the matching layer for a 1D absorber) can be used for sphere (3D) and cylinder (2D) with light-absorbing shells. The core-shell objects are surrounded by transparent mediums that is the same as the cores. The simulated photoacoustic signals for the sphere with the core *r*_c_ and the shell 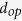
are shown in Fig. 5(a) for the long pulse regime (*τ* ≫ 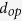/c). The photoacoustic signals are normalized to the signal amplitude for *r*_*c*_ = 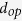. For a uniform volumetric heating energy *H* = *H*_0_(t), the photoacoustic amplitudes significantly increase with the core radius *r*_c_. Also, the photoacoustic pulses have the bipolar waveforms (the time-derivative of the temporal optical intensity profile)^11^. Interestingly, the acoustic signal for *r*_*c*_ = 25 *d*_*abs*_ is two orders of magnitude higher than that for *r*_c_/*t*_abs_ = 1. However, such difference cannot be solely explained by the cancellation effect, but rather by the combined effect of the PA cancellation and the 1/*r* amplitude decay of spherical acoustic waves. With larger radius *r*_*c*_, the photoacoustic source is much closer to the observation location *r*_obs_, thus reducing the acoustic decay. To evaluate only the contribution of the cancellation effect, the photoacoustic signals are re-calculated by using the heating energy *H*(t) scaled with 1/*r*, i.e., *H*(*r*, *t*) = *H*_0_(*t*)/*r*. As shown in Fig. 5(b), the scaled photoacoustic amplitudes increase with the core radius. Also, the inward wave is completely separated from the original outward wave at *r*_c_ = 100 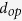. The time delay between the two pulses is identified to correspond to an acoustic transit time across the core diameter (2*r*_c_/*c*). Moreover, the interaction of the two pulses observed here for different time-lag are similar to that for a 1D absorber in the near field (see Supplementary Note 6 online).

Figure 5(c) shows the simulated acoustic signals for the cylinder with the transparent core and the light-absorbing shell. Here, the heating energy is scaled by 1/ *r*^0.5^ for the acoustic decay of cylindrical waves. Similar to those for the sphere, the photoacoustic signals show bipolar waveforms, but with higher positive amplitude than negative one (which follows the one-half time-derivative of the optical radiation intensity)^11^. It is clear that the amplitude increases with the core radius *r*_c_. And the two waves are completely separated at a core radius of *r*_c_ = 100 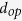, which gives a sufficient delay to the inwardly-propagating wave. The delayed wave has a bipolar waveform due to a π/2 Gouy phase shift, while the original out-going wave shows a mono-polar waveform. It is noteworthy that the amplitude increase by the matching core is much smaller than that of the sphere. This is because the delayed wave cannot completely cancel the original out-going wave in the 2D case. Thus, the transparent matching core is less effective in the cylinder case than in the sphere case. For further comparison with the sphere, the out-coupling efficiency is plotted in Fig. 5(d). Unlike the sphere case with the monotonic increase, the cylinder case exhibits a peak (*η* >1) at a core radius of *r*_c_ = 30 *d*_abs_. At that radius (its corresponding time delay is 1/4*cτ*), the two positive peaks of the two waves are overlapped, leading to the highest amplitude.

### Summary

We have systematically investigated the cancellation effect that is responsible for significantly low longitudinal photoacoustic (PA) amplitude for the soft boundary. In typical measurement, the two waves with opposite polarity are so close to each other that each wave has hardly been identified. Unlike thick, impedance-mismatched transparent coverings, a thin impedance-matched layer is used to effectively control the overlap of two symmetric photoacoustic pulses, and thus to minimize the cancellation effect. Despite the addition of the thin layer, photoacoustic waveforms are preserved, which could provide with tunability of photoacoustic waveforms.

The cancellation effect is influenced by the optical penetration depth. For small optical penetration depths, the cancellation effect is significant such that the matching layer is very effective in recovering the PA amplitude. However, for large optical penetration depths, the cancellation effect is less significant. This is because large optical penetration depths result in reduced overlap due to increased time delay between the two pulses.

The cancellation effect observed in the planar absorber can be similarly seen in the other dimensions including 2D and 3D. Unlike the 1D and 3D cases, the cylinder case shows lower phase cancellation, since the reflected pulse has a bipolar waveform (a *π*/2 phase shift) that is different from a monopolar waveform of the original outward wave. Moreover, the phase overlap between the bipolar and monopolar waveforms gives an idea of why the resulting wave shows the asymmetric bipolar waveform consisting of the positive amplitude twice larger than the negative one. This finding could offer further understanding of photoacoustic generation and an idea for designing photoacoustic contrast agents.

## Methods

### Photoacoustic generation and detection

By irradiating a nanosecond pulsed laser (Surelite I-20, Continuum, Santa Clara, CA), photoacoustic signals are generated. The laser with a repetition rate of 20 Hz can deliver a laser pulse of 6 ns FWHM (Full Width and Half Maximum). The photoacoustic signals are measured in the far field by using a home-made PVDF detector with 28 μm in thickness (estimated bandwidth ~40 MHz). The signals are monitored by using a digital oscilloscope (WaveSurfer 432, LeCroy, Chestnut Ridge, NY). For the far-field measurement, the laser beam with 5 mm in diameter is reduced to 1 mm by using an aperture of 1 mm in diameter. Also, to fulfill the far-field requirement, the distance between the detector and the source is much larger than the illuminated area.

### Planar samples for photoacoustic generation

The metal absorbers are composed of thin Cr layer (100 nm) and PDMS layer (20 μm). The metal layer is deposited by a sputter tool. For an experiment of varying water layer thickness, a polymer absorber is used, which consists of a carbon nanotube (CNT)-polymer composite (20 μm thickness), where CNTs are embedded in polydimethylsiloxane (PDMS) elastomer^22^.

### Photoacoustic generation simulation

Simulation is carried out by numerically solving the heat conduction equations and pressure wave equation through the finite element method (the Courant number CFL <0.05; COMSOL Multiphysics 4.3b) (see Supplementary Note 2 online for details).

## Additional Information

**How to cite this article**: Lee, T. *et al.* Out-coupling of Longitudinal Photoacoustic Pulses by Mitigating the Phase Cancellation. *Sci. Rep.* **6**, 21511; doi: 10.1038/srep21511 (2016).

## Supplementary Material

Supplementary Information
