# Supplementary material for: Out-coupling of Longitudinal Photoacoustic Pulses by Mitigating the Phase Cancellation
Source: Sci Rep. 2016 Feb 12;6:21511. doi: 10.1038/srep21511 (PMC4751463; doi:10.1038/srep21511)
Supplement: Supplementary Information [file srep21511-s1.pdf]

Supplementary Information

Out-coupling of Longitudinal Photoacoustic Pulses by Mitigating the Phase Cancellation

Taehwa Lee,[1] Qiaochu Li, [2] and L. Jay Guo[1,2,*]

[1]Department of Mechanical Engineering, University of Michigan, Ann Arbor, MI48109, USA
[2]Department of Electrical Engineering and Computer Science, University of Michigan, Ann Arbor, MI48109, USA

Supplementary Note1: Effect of acoustic impedance on out-coupling of photoacoustic pulses

For thick transparent coverings, there is a considerable acoustic reflection (s2), which partially cancels the pulse (s1), as illustrated in Fig. S1. Thus, the interference cannot be completely eliminated. Also, the reflected pulse from the free surface (s4) will not overlap with the main pulse (s1). Specifically, for thick transparent coverings, the effect of mechanical properties on the two pulse interference is estimated, as shown in the figure below. As the acoustic impedance of transparent coverings (Z_m) increases to that of light absorbers (Z), the out-coupled pressure amplitude is maximized because of minimized reflection (s2). For example, for liquid transparent coverings on metals ($Z_m/Z < 0.1$), the cancellation effect is significant. In our study, we use acoustically matched transparent coverings, leading to negligible reflection. Also, unlike thick transparent coverings, thin optimized transparent matching layer in our study can allow the reflected pulse (s4) to constructively interfere with the pulse (s1), leading to much larger pressure amplitude.

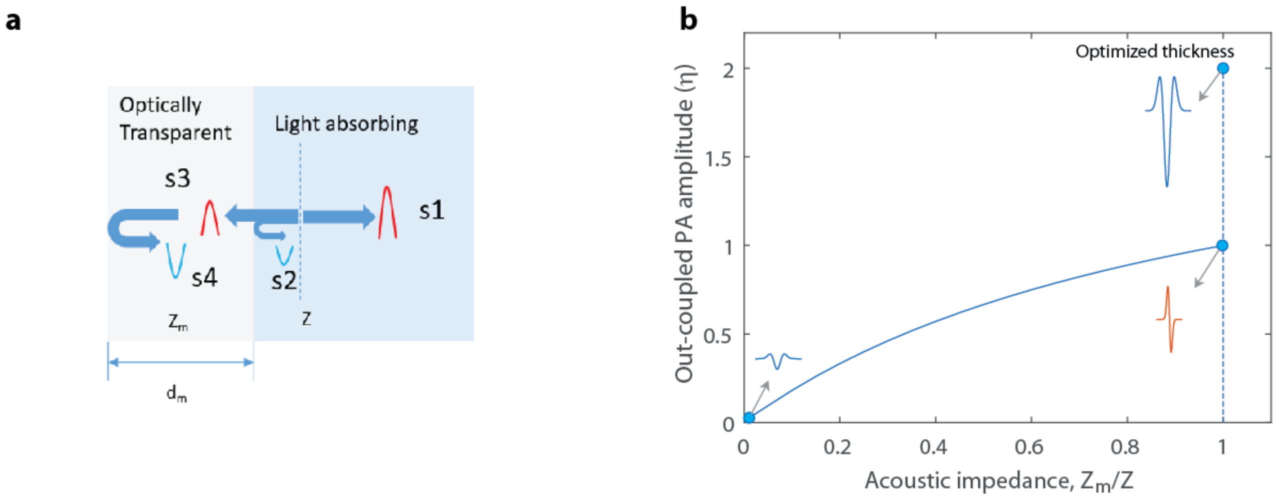

Fig. S1 (a) Schematic diagram of reflection and transmission of photoacoustic (PA) pulses. (b) Effect of acoustic impedance of transparent covering on out-coupling of PA pulses

Supplementary Note2: Simulation of photoacoustic generation

Photoacoustic signals are calculated by numerically solving the heat conduction equation and pressure wave equation through the finite element method (the Courant number CFL < 0.05; COMSOL Multiphysics 4.3b). The heat conduction equation yields the time-dependent temperature field $T(\vec{r},t)$ induced by pulsed-laser heating , which is represented by

$$\rho C_P \frac{\partial T}{\partial t} = \nabla(k\nabla T) + H(\vec{r},t), \tag{1}$$

where ρ is the density, C_P is the specific heat capacity at constant pressure, k is the thermal conductivity, and $H(\vec{r},t)$ is the volumetric non-radiative heat generation due to light absorption (W/m^3). The above equation is based on the assumption that adiabatic heating induced by compressive pressure are negligible when C_P is almost identical to the specific heat capacity at constant volume C_V [1]. With the calculated temperature from the equation (1), the pressure field $p(\vec{r},t)$ in a liquid can be determined by solving a wave equation for pressure represented by

$$\left[\nabla^2 - \frac{1}{c^2}\frac{\partial^2}{\partial t^2}\right] p(\vec{r},t) = Q_{aco} = -\rho\beta\frac{\partial^2 T}{\partial t^2}, \tag{2}$$

where c is sound speed, Q_{aco} is an acoustic source term modeled by thermoelastic mechanism, and β is the thermal expansion coefficient.

Supplementary Note3: Analytical solution for different matching layer

By using the equation (1) for negligible heat conduction (i.e., $\nabla(k\nabla T) = 0$), which corresponds to $\rho C_p \frac{\partial T}{\partial t} = H(\vec{r},t)$, the equation (2) can be alternatively represented by [2, 3]

$$\left[\nabla^2 - \frac{1}{c^2}\frac{\partial^2}{\partial t^2}\right] p(\vec{r},t) = -\frac{\beta}{C_P}\frac{\partial}{\partial t} H(\vec{r},t), \tag{3}$$

This is valid when the heat does not diffuse over the spatial extent of the acoustic pulse defined by $l_{aco} = c\tau_{aco}$, where τ_{aco} is the acoustic pulse duration. In other word, heat diffusion length during the acoustic pulse duration ($l_{th} = \sqrt{\alpha\tau}$, thermal diffusivity $\alpha = k/\rho C_P$) is much smaller than l_{aco}. The one-dimensional form of the equation (3) is typically used and can be expressed by

$$\left[\frac{\partial^2}{\partial z^2} - \frac{1}{c^2}\frac{\partial^2}{\partial t^2}\right] p(z,t) = -\frac{\beta}{C_P}\frac{\partial}{\partial t} H(z,t), \tag{4}$$

where *z* is the spatial variable. The one dimensional equation in a liquid also holds for an isotropic solid

when the sound speed c is substituted by the longitudinal sound speed c_L and the thermal expansion coefficient β is replaced by the effective thermal expansion coefficient $\beta_{effective} = \beta\left(1-4c_T^2/3c_L^2\right)$, where c_T is the transverse sound speed [4].

Applying the Green's function approach to the one-dimensional wave equation (4) yields the general solution for pressure, which is represented by

$$p(z,t) = \frac{\beta c}{2C_P}\int_{-\infty}^{\infty} dz' g(z,t\,|\,z',t) H\left(z', t-\frac{z-z'}{c}\right), \tag{5}$$

where $g(z,t\,|\,z',t)$ is the Green's function for an infinite medium. The appropriate Green's function for $z > z'$ is $g(z,t\,|\,z',t) = 2\pi c u\left((t-t')-(z-z')/c\right)$, where u is the Heaviside function. Then, the excited photoacoustic pulses has the form of $p(z,t) = \frac{\beta c}{2C_P}\int dz' H(z', t - \frac{z-z'}{c})$.

The one-dimensional Green's function above can be modified to take into account the reflected pulse from the interface, which is produced simultaneously with the original forward-propagating pulse. The modified Green's function can be represented by the sum of two one-dimensional Green's functions $g(z,t\,|\,z',t) + R\cdot g(z,t\,|-z',t)$, where R is the acoustic reflectance (-1 for a sound soft boundary and +1 for sound hard boundary) [2]. Thus, the photoacoustic signals for different boundaries can be represented by [2, 5]

$$p(z,t) = \frac{\beta c}{2C_P}\int_{-\infty}^{\infty} dz'\left[H\left(z', t-\frac{z-z'}{c}\right) + R\cdot H\left(z', t-\frac{z+2d_m+z'}{c}\right)\right], \tag{6}$$

where d_m is the thickness of the additional layer between the reflecting boundary and the origin (the reflecting boundary is located at $z=-d_m$). Here, the heating function $H(z',t)$ can be expressed as product of temporal and spatial heating functions, $f(t)$ and $g(z')$ by $H(t,z') = I_0 f(t) g(z')$ (W/m^3), where I_0 is the peak intensity (W/m^2), $f(t)$ is related to energy fluence F through $F = \int I_0 f(t)\,dt$ (J/m^2), and $g(z')$(1/m) is normalized for satisfying $\int g(z')dz' = 1$. With the retarded time ($\tau = t - z/c$), the first pulse of the equation (6) can be specified in mathematics by the cross-correlation integral $\int f(c\tau + z') g(z') dz'$, while the second reflected pulse can be by the convolution integral $\int f(c\tau - z') g(z') dz'$.

Supplementary Note4: Intrinsic time delay

The pulse-to-pulse delay time (dashed line) is compared with time delay resulting from the matching layer (solid line), as shown in Fig. S2. There is a constant offset, which is identical to the time delay corresponding to the optical penetration depth ($d_{op} = 1/\alpha$, where α is the light absorption coefficient). The time delay can hinder complete phase overlap, even without matching layer.

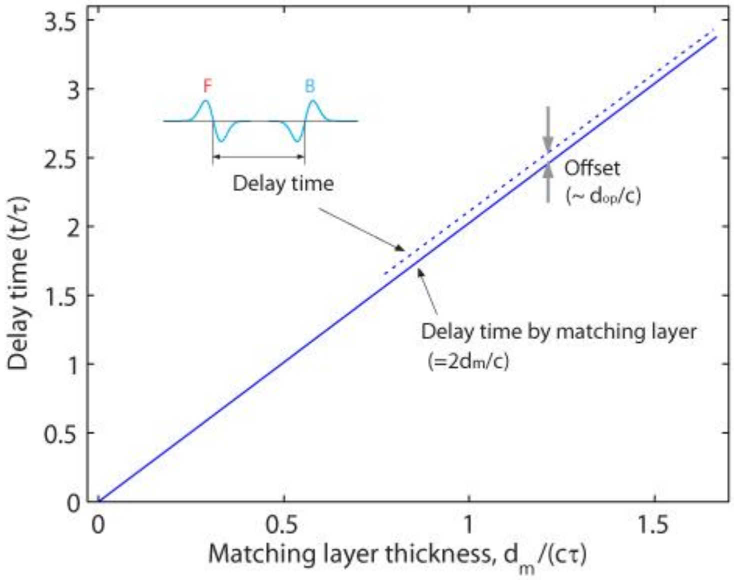

Fig. S2 Comparison between the pulse-to-pulse time delay (dashed line) and the time delay by matching layer (solid line) $(= 2d_m / c)$. For $d_m/c\tau$ >1, the two pulses are completely separated, thus showing apparent pulse-to-pulse delay, which is slightly larger than the delay time. The difference is caused by the optical penetration.

Supplementary Note5: The derivation of out-coupled PA amplitude for the long pulse regime

Photoacoustic signals subject to the boundary conditions can be obtained by Laplace transform approach [4], which can be represented by

$$p_H(t) = \frac{\beta c I_0}{C_P} f(t), \text{ and } \ p_S(t) = \frac{\beta I_0}{\alpha C_P} \frac{df(t)}{dt}. \qquad (7)$$

By using the Gaussian temporal pulse $f(t) = \exp\left(-t^2 / \tau_l^2\right)$ and the maximum value of the time-derivative $\left|\frac{df(t)}{dt}\right|_{\max} \sim \frac{1}{\tau_l}$, the amplitude ratio of the two signals (7) is expressed by

$$\frac{P_{S,0}}{P_{H,0}} \sim \frac{1}{\alpha c} \cdot \frac{1}{\tau_l}.$$

The photoacoustic signal for the radiation boundary is half of that for the hard boundary, i.e., $P_{R,0} = 0.5 P_{H,0}$. Thus, the out-coupled PA amplitude for the long pulse regime can be represented by

$$\gamma = \frac{P_{S,0}}{P_{R,0}} = c_1 \frac{1}{\alpha c} \cdot \frac{1}{\tau_l} = c_1 \frac{\left(d_{op} / c\right)}{\tau_l}, \qquad (8)$$

where d_{op} is the optical penetration depth ($1/\alpha$), c_1 is the constant. Note that is represented by the two time scales: the intrinsic time delay due to optical penetration depth and the acoustic pulse duration (τ_l). The constant c_1 is calculated to be approximately 2.84.

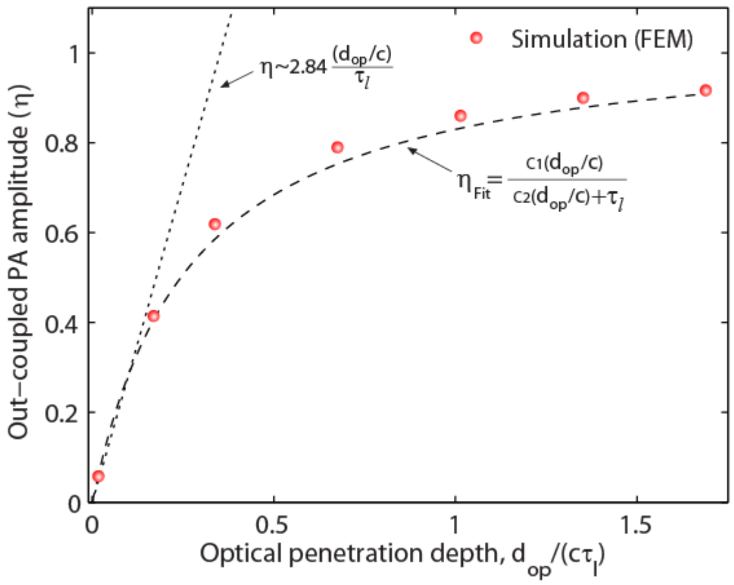

Fig. S3 Comparison between the pulse-to-pulse time delay (dashed line) and the time delay by matching layer (solid line).

***c.f.*: The alternative derivation of equation (7)**

Both the cross-correlation and convolution integrals, representing the first and second pulses in the equation (6), can be approximated by the temporal heating function $f(\tau)$ for the long pulse regime, where the temporal duration of the heating function $f(t)$ is longer than the acoustic transit time across the heated zone localized at $z = 0$ (i.e., $g(z') \approx \delta(z')$), Thus, for sound hard (p_H) and sound soft (p_S) boundary conditions, the photoacoustic signals ($d_m = 0$) can be represented by

$$p_H(\tau) = \frac{\beta c I_0}{2C_P}\left\{ f(\tau) + f(\tau - \tau_{abs}) \right\},$$

$$p_S(\tau) = \frac{\beta c I_0}{2C_P}\left\{ f(\tau) - f(\tau - \tau_{abs}) \right\}, \tag{9}$$

where τ_{abs} is the time delay of $2l/c$ due to the finite length of the heated depth specified by $l = 1/\alpha_{op}$ for $g(z) = \alpha_{op}\exp(-\alpha_{op} z)$, where α_{op} is the light absorption coefficient. Since τ_{abs} is very small, the photoacoustic signals can be further simplified by

$$p_H(\tau) \cong \frac{\beta c I_0}{C_P} f(\tau)$$

$$p_S(\tau) = \frac{\beta c I_0 \tau_{abs}}{2C_P} \frac{f(\tau) - f(\tau - \tau_{abs})}{\tau_{abs}} \cong \frac{\beta I_0}{\alpha C_P} \frac{df(\tau)}{d\tau}. \tag{7}$$

Supplementary Note6: The out-coupled PA amplitude for a planar absorber in the near field

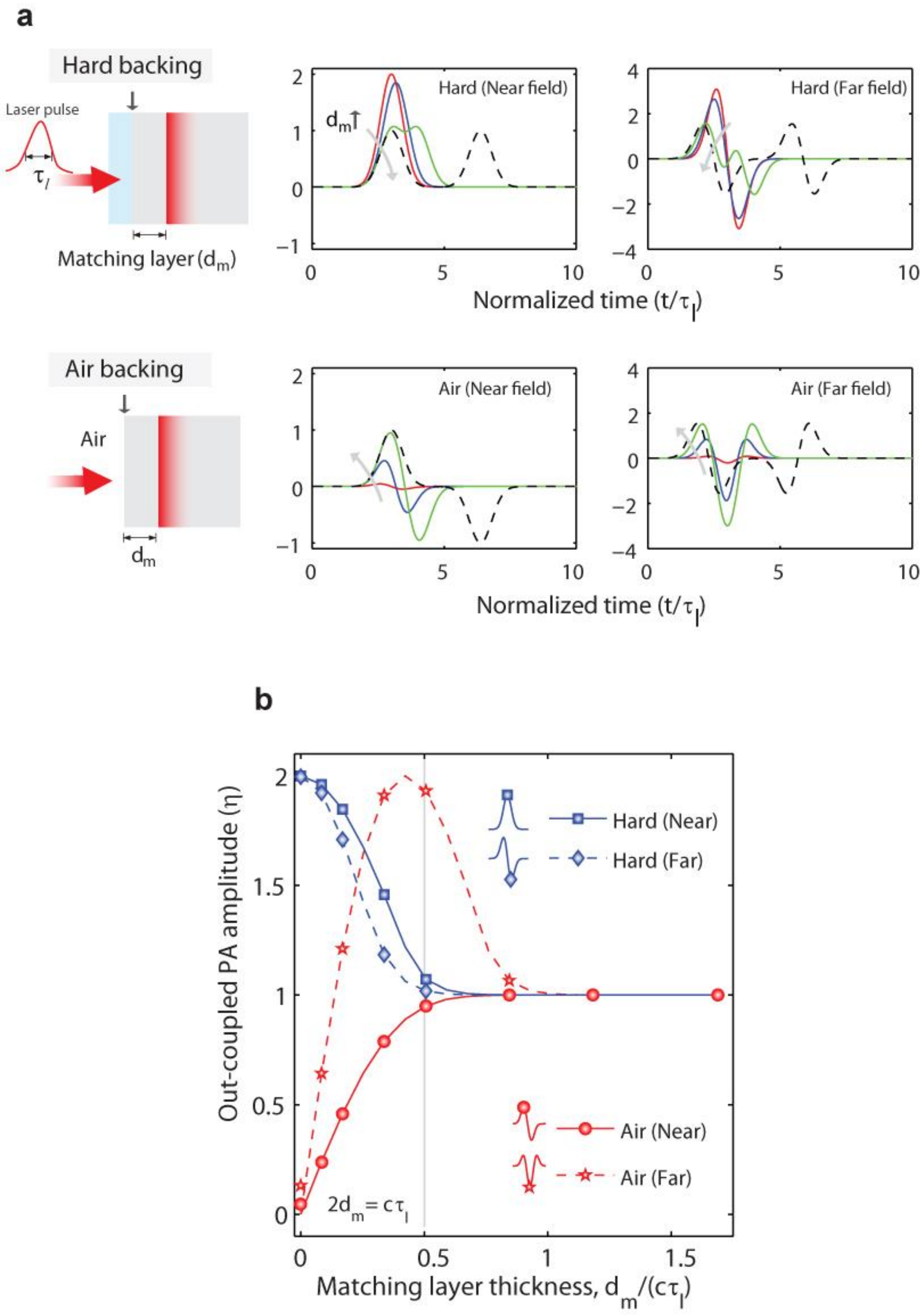

Fig. S4 (a) Comparison between the pulse-to-pulse time delay (dashed line) and the time delay by matching layer (solid line). (b) The out-coupled PA amplitudes for hard and soft boundary in the near and far fields.

Supplementary Note7: The effect of absorption profile on the out-coupled PA ampltiude

To gain further insight into phase cancellation, effect of light absorption profile on the out-coupling efficiency is examined. Three different light absorption profiles used are illustrated in Fig. S5(a): linearly decaying intensity (a1), constant intensity (a2), and linearly increasing intensity (a3). Total optical energy for all cases remains constant. The calculated out-coupling efficiency as a function of d_{op} is plotted in Fig. S5(b) for no the matching layer. For thinner layers ($d_{op} / c\tau_l < 0.1$), regardless of absorption profiles, significantly lower out-coupling efficiency is. For moderate depth d_{op}, however, the linearly increasing intensity has higher efficiency than the others. Furthermore, as expected, a light absorption profile decaying exponentially from an interface can have even smaller efficiency than the linearly decaying intensity. This absorption profile dependence can be explained by considering that photoacoustic signal for a thick absorbing layer results from the combined amplitudes from the virtually divided thin layers [see the inset of Fig. 5(b)], each with different out-coupling efficiency depending on the distance from the interface. This indicates that for higher out-coupling efficiency, higher light intensity should be applied to the layers far from the air-backed interface. Also, direction of light irradiation is important, as it can determine absorption profile. Thus, light illumination from the air side, which usually produces an absorption profile of exponential decay, can show lower photoacoustic amplitude than from the other side.

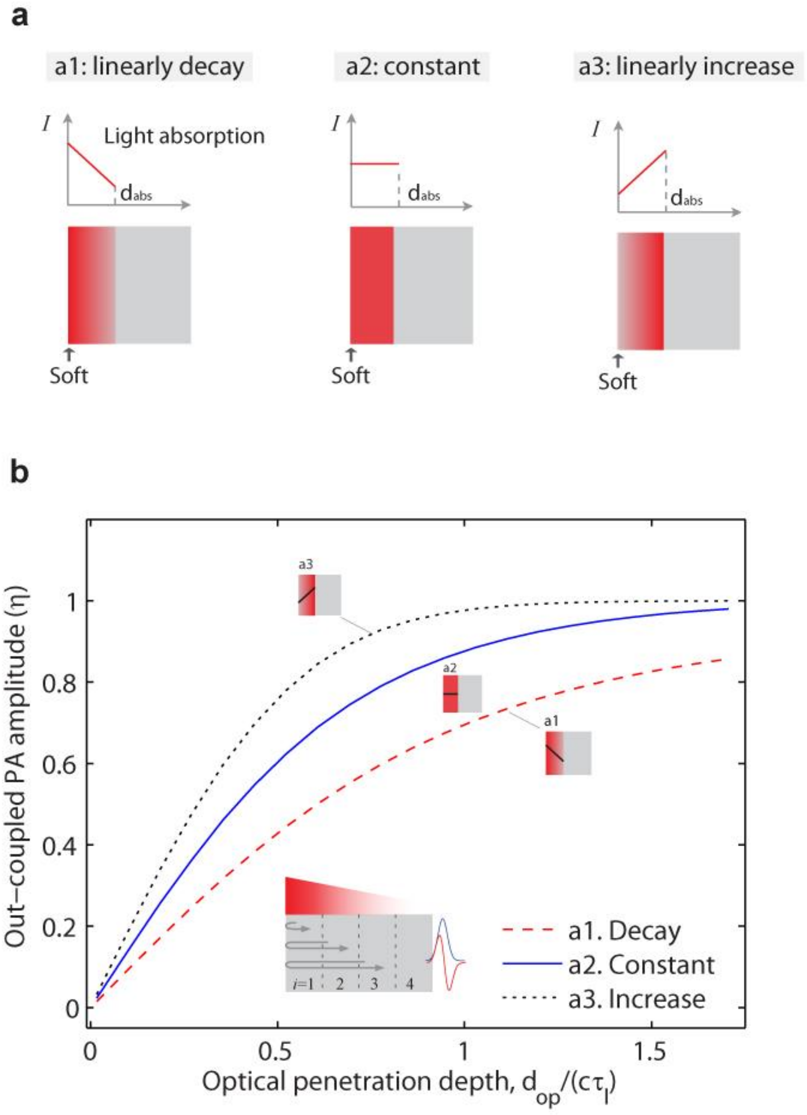

Fig. S5 The effect of light absorption profile on the out-coupled PA amplitude without the matching layer. (a) Illustration of three light absorption profiles (a1: linearly decay from the interface, a2: constant light absorption, a3: linearly increase from the interface). (b) The out-coupled PA amplitudes of the three profiles (decay: dashed line, constant: solid line, increase: dotted line)

[1] L.V. Wang. Photoacoustic Imaging and Spectroscopy, CRC Press, 2009.

[2] G.J. Diebold, T. Sun, M.I. Khan. PHOTOACOUSTIC MONOPOLE RADIATION IN 1-DIMENSION, 2-DIMENSION, AND 3-DIMENSION, Physical Review Letters 67 (1991) 3384-3387.

[3] M.H. Xu, L.H.V. Wang. Photoacoustic imaging in biomedicine, Review of Scientific Instruments 77 (2006).

[4] V.E. Gusev, A.A. Karabutov. Laser Optoacoustics, American Institute of Physics, New York, 1993.

[5] G.J. Diebold. Photoacoustic waves at reflecting interfaces, Review of Scientific Instruments 74 (2003) 801-804.

[6] D. Kim, M. Ye, C.P. Grigoropoulos. Pulsed laser-induced ablation of absorbing liquids and acoustic-transient generation, Applied Physics a-Materials Science & Processing 67 (1998) 169-181.
